# Performance Evaluation of the Schistoscope 5.0 for (Semi-)automated Digital Detection and Quantification of *Schistosoma haematobium* Eggs in Urine: A Field-based Study in Nigeria

**DOI:** 10.4269/ajtmh.22-0276

**Published:** 2022-10-17

**Authors:** Brice Meulah, Prosper Oyibo, Michel Bengtson, Temitope Agbana, Roméo Aimé Laclong Lontchi, Ayola Akim Adegnika, Wellington Oyibo, Cornelis Hendrik Hokke, Jan Carel Diehl, Lisette van Lieshout

**Affiliations:** ^1^ Department of Parasitology, Leiden University Medical Center, Leiden, The Netherlands;; ^2^ Industrial Design Engineering, Delft University of Technology, Delft, The Netherlands;; ^3^ Mechanical, Maritime and Material Engineering, Delft University of Technology, Delft, The Netherlands;; ^4^ Centre de Recherches Médicales des Lambaréné, CERMEL, Lambaréné, Gabon;; ^5^ Centre for Malaria Diagnosis, NTD Research, Training & Policy/ANDI Centre of Excellence for Malaria Diagnosis, University of Lagos, Lagos, Nigeria;; ^6^ Institut fur Tropenmedizin, Universitat Tubingen, Tubingen, Germany;; ^7^ German Center for Infection Research (DZIF), partner site Tübingen, Germany

## Abstract

Conventional microscopy is the standard procedure for the diagnosis of schistosomiasis, despite its limited sensitivity, reliance on skilled personnel, and the fact that it is error prone. Here, we report the performance of the innovative (semi-)automated Schistoscope 5.0 for optical digital detection and quantification of *Schistosoma haematobium* eggs in urine, using conventional microscopy as the reference standard. At baseline, 487 participants in a rural setting in Nigeria were assessed, of which 166 (34.1%) tested *S. haematobium* positive by conventional microscopy. Captured images from the Schistoscope 5.0 were analyzed manually (semiautomation) and by an artificial intelligence (AI) algorithm (full automation). Semi- and fully automated digital microscopy showed comparable sensitivities of 80.1% (95% confidence interval [CI]: 73.2–86.0) and 87.3% (95% CI: 81.3–92.0), but a significant difference in specificity of 95.3% (95% CI: 92.4–97.4) and 48.9% (95% CI: 43.3–55.0), respectively. Overall, estimated egg counts of semi- and fully automated digital microscopy correlated significantly with the egg counts of conventional microscopy (*r* = 0.90 and *r* = 0.80, respectively, *P* < 0.001), although the fully automated procedure generally underestimated the higher egg counts. In 38 egg positive cases, an additional urine sample was examined 10 days after praziquantel treatment, showing a similar cure rate and egg reduction rate when comparing conventional microscopy with semiautomated digital microscopy. In this first extensive field evaluation, we found the semiautomated Schistoscope 5.0 to be a promising tool for the detection and monitoring of *S. haematobium* infection, although further improvement of the AI algorithm for full automation is required.

## INTRODUCTION

Schistosomiasis is a neglected tropical disease affecting approximately 250 million people, and more than 700 million people are at risk of infection.^1^ Sub-Saharan Africa shares the greatest burden of this disease,^2^ and preschool and school-age children are the most affected. It is a parasitic worm infection of poverty, leading to chronic disease and significant disability-adjusted life years lost.^3^ Several *Schistosoma* species are known to affect humans. Urogenital schistosomiasis is caused by *S. haematobium*, and *S. mansoni* is the major species causing intestinal disease. *S. haematobium* infections are most prevalent in Africa, affecting the urogenital system with hematuria, bladder and kidney failure as the main complications and genital schistosomiasis presentations such as vaginal discharge and postcoital bleeding in women and hematospermia in men.^3^^,^^4^ Chronic infections can lead to miscarriage and infertility and may facilitate infection with sexually transmitted diseases, including HIV.^4^

The prevailing strategy to control and eliminate this disease is a comprehensive integrated program of mass drug administration (MDA) with praziquantel, water, sanitation, and hygiene (WASH); snail vector control; and a multisectoral approach to diagnostic monitoring and evaluation.^5^ The diagnosis of *S. haematobium* infection typically involves the detection of eggs in urine by conventional light microscopy. Counting the number of eggs seen per 10 mL of urine is commonly done to indicate the intensity of infection in a target population,^3^^,^^5^ which is relevant for the purpose of monitoring and evaluation. However, the need for expert laboratory personnel, basic laboratory infrastructure, and a permanent power supply limits the use of conventional light microscopy in endemic resource-limited settings. In addition, in areas where laboratory infrastructure is inadequate, the ratio of trained personnel to sample analysis is often very low, resulting in a high workload per technician and above threshold eye exposure to the microscopy light source, causing visual health complications.^6^^,^^7^ Therefore, there is a need for innovative, and preferably easy-to-use, diagnostics that will suit endemic resource-limited settings to diagnose infections and complement control and elimination efforts.

During the past decade innovative optical diagnostic devices, with or without artificial intelligence (AI), have been developed for the detection of *S. haematobium* eggs.^8^^–^^15^ Although several of these devices scan through samples and save digitalized images for manual identification of *Schistosoma* spp.,^8^^–^^12^ only a few have an integrated AI program for automated detection.^13^^–^^15^ To our knowledge, only four of these devices have been field validated using samples from a *Schistosoma*-exposed population,^9^^–^^12^ and only the Newton Nm1 microscope has been marketed commercially as a portable field microscope, although without a fully automated AI application.^12^ This limited validation highlights the technical challenges that are faced to transition working prototypes to commercialized and field applicable devices. Also, most studies have used only a small, often nonrandomly selected, number of clinical samples to validate the diagnostic devices. Hence, there is a clear need for more extensive field-based studies.

**Figure 1.**
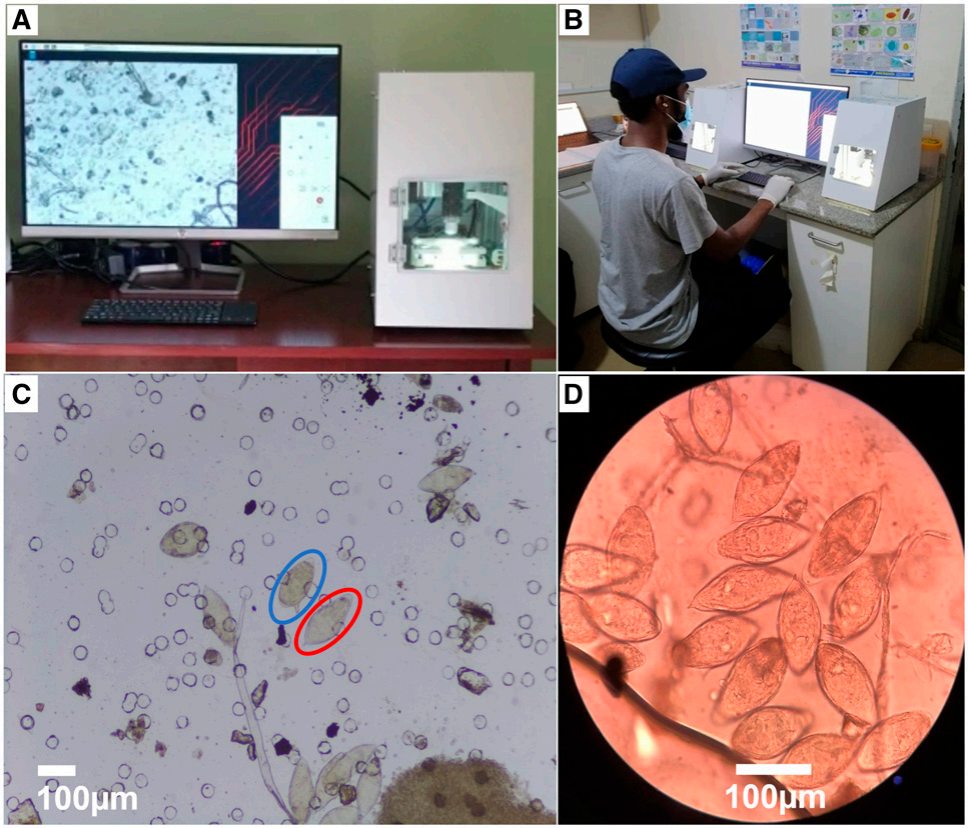
(A) Schistoscope 5.0 device (right) connected to a computer monitor (left), showing an image of a digitally screened sample. (**B**) Schistoscope 5.0 operated by a laboratory technician in the field. (**C**) Digital image of a urine filtered membrane showing several *Schistosoma* eggs captured with the Schistoscope 5.0 (4× objective). The red circle indicates a *S. haematobium* egg, the blue circle indicates a *S. mansoni* egg. (**D**) Image of a urine filtered membrane with several *S. haematobium* eggs captured by a camera attached to a conventional microscope (10× objective). This figure appears in color at www.ajtmh.org.

The Schistoscope device (version 5.0) is a low-cost digital microscope (Figure 1A and B) that has gone through five design iterations in an ongoing process of co-creation including different potential stakeholders. In its current form, it can function either as a semiautomated or AI integrated fully automated digital microscope to detect and quantify *S. haematobium* eggs.^16^^,^^17^ In a recent proof-of-principle study, the device and its AI algorithms were trained successfully with phosphate buffer saline and urine samples that were spiked with *S. haematobium* eggs obtained from a laboratory maintained parasite life cycle and a limited number of clinical samples.^18^ This led to the conclusion that the Schistoscope was ready for further validation. The aim of the current study is to evaluate the performance of the Schistoscope 5.0 as a semi- and fully automated digital microscope for the detection and quantification of *S. haematobium* eggs in a prospective study design under field conditions. For this purpose, urine samples were collected in a rural area in Nigeria and filtered, and each membrane filter was independently examined locally by conventional microscopy and the Schistoscope 5.0.

## METHODS

### Ethical considerations.

This study was done in collaboration with the Schistosomiasis Program of the Neglected Tropical Diseases Department, Federal Ministry of Health, Abuja, and embedded in an ongoing, cross-sectional community-based survey in collaboration with the Public Health Department in charge of the MDA of praziquantel in the Federal Capital Territory (FCT), Nigeria. The ethical approval for this study was obtained from the FCT Health Research Ethics Committee in Abuja, Nigeria (reference no. FHREC/2019/01/73/18-07-19). Written consent from adults and from parents or legal guardians of children and teenagers was obtained before sample collection from persons willing to participate through their signatures or thumbprints. Confidentiality and anonymity of results were ensured by assigning unique codes to samples. According to the local standard operational procedures, all participants with detectable hematuria (discussed subsequently) were considered *S. haematobium* positive and therefore treated with praziquantel (40 mg/kg of body weight). The local health authorities have been informed of the outcome of the study, and all participants have been offered (re)treatment where appropriate.

### Study design and population.

This cross-sectional and longitudinal study was carried out in August–September 2021 in two area councils in FCT, Abuja, Nigeria (geographic coordinates: 9.0618° N latitude, 7.4221° E longitude and 8.950833° N latitude, 7.076737° E longitude). The FCT is the third highest endemic state for schistosomiasis in Nigeria.^19^ In total, 14 communities from these two area councils were visited, where preschool, school-age children and adults were allowed to participate. Strategic advocacy and engagement with community leaders in the study area preceded the sample collection at the communities studied.

### Sample collection and processing.

**Figure 2.**
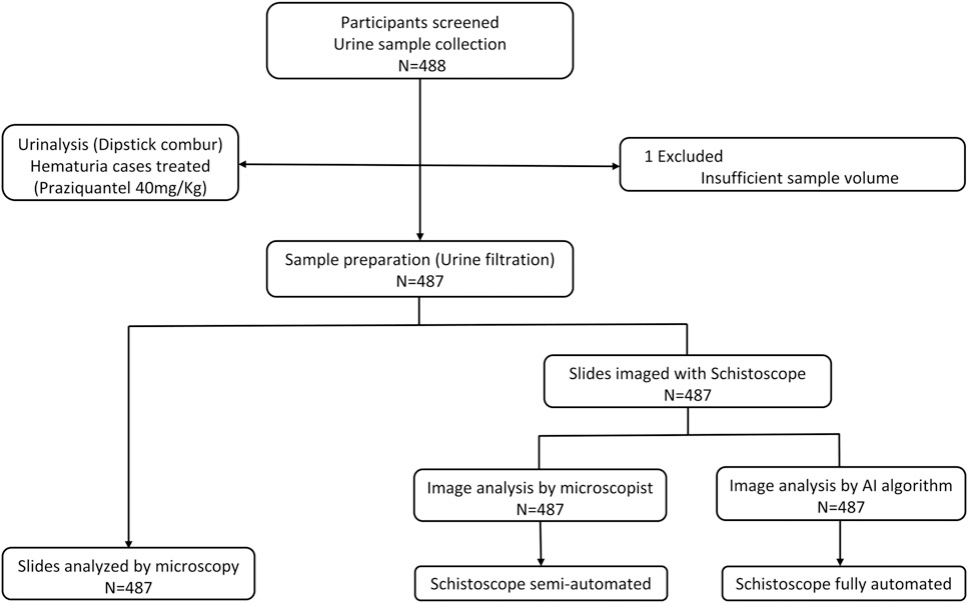
Flow chart of urine sample collection and analysis comparing conventional microscopy with semiautomated and fully automated digital microscopy.

Figure 2 depicts the flowchart of sample collection. Briefly, a sterile 20-mL universal container with a unique identification code was given to those who consented to participate with the request to collect a urine sample between 11:00 am and 13:00 pm. Dipstick (Combur 10-Test M Roche Mannheim, Germany) urinalysis was performed on site according to the manufacturer’s instructions. Of those who were confirmed as positive by conventional urine microscopy, 50 were randomly selected and asked to provide an additional sample 10 days after baseline screening. This small-scale posttreatment evaluation was done to examine whether drug treatment could influence the performance of the Schistoscope 5.0, possibly via praziquantel-induced changes in egg morphology.^20^^,^^21^

All urine samples were transported to the laboratory of the Department of Public Health, Abuja, FTC, within 2 hours of sample collection and prepared for microscopy by urine filtration.^22^ Urine samples were homogenized, and 10 mL of urine was obtained with a syringe and pressed through a filter membrane (diameter 13 mm; pore size 30 µm; Whatman International Ltd., Maidenstone, UK). The filter membrane was then placed on a standard microscope glass slide, and a cover slip was placed over the membrane to keep the filter moist. Each slide was viewed under a standard microscope and the Schistoscope.

### Description of the Schistoscope 5.0.

The Schistoscope 5.0 (Figure 1) is a low-cost automated slide-scanner digital microscope that can be supported with AI algorithms for image processing.^18^ The system is composed of custom-designed optical bright-field illumination, three-axis movement (X, Y, Z), and electronic and computing modules. The illumination module comprises a bright white light-emitting diode and condenser lenses to generate uniform illumination. The custom three-axis motorized stage provides a step resolution of 2.5 µm on all three axes. A custom printed circuit board is used to control all three motors and the illumination. The on-board computer is a Raspberry Pi 4B connected to a Raspberry Pi HQ camera that has a pixel size of 1.55 µm and an image resolution of 2028 × 1520 pixels. The current study used a 4× microscope objective that provides an experimental resolution limit of 3.26 μm,^18^ which is sufficient to resolve *S. haematobium* eggs (Figure 1C). The device runs on mains electricity and does not have a built-in battery. Dedicated software with a graphical user interface was developed and installed on the device’s onboard computer for easy user interaction and control of the device. The software comprises a simple autofocus procedure and an algorithm to scan the complete filter membrane and capture each field of view as an image. It takes 12 minutes to scan and capture 117 images of an entire 13-mm filter membrane. Additional analysis of the captured images, including counting the number of eggs, takes approximately 5 minutes on average per filter when done either manually or by AI. Captured images are stored in folders by their sample identification code. Semiautomated analysis can be done via a connected computer monitor, or automated analysis can be done on an external computer. Further development is ongoing to enable automated processing and analysis on the device itself.

### Detection of *S. haematobium* eggs by microscopy and the Schistoscope.

Slides were examined immediately after preparation. The order of examination was randomized, resulting in approximately half of the slides being first analyzed by conventional microscopy and then imaged with the Schistoscope 5.0 and the other half analyzed in the opposite sequence.

For conventional light microscopy, slides were analyzed using a 10× objective on an Olympus (Tokyo, Japan) CX22RFS1 microscope (Figure 1D). Two microscopists independently examined each slide for the detection and quantification of *S. haematobium* eggs with results blinded from each other. The average of egg counts from both microscopists was computed as the final result. Discrepancies of more than 20% between both microscopy readings were resolved by a third independent microscopy reading, of which an average between two closest among the three readings was considered.

The imaging procedure of the Schistoscope included manual counting of the eggs seen on the images, which was done in the field by a fourth microscopist who was blinded from the results of the conventional light microscopy. The images were also uploaded to a cloud server (Google Colaboratory; https://colab.research.google.com) for remote access and AI analysis. For quality control of the manual analysis of the captured images, 10% of the images were randomly selected and reexamined by an independent senior microscopist, but because this showed no significant differences from the original manual readings, these data are not further considered. Data from the two independent microscopists, the manual reading, and the AI analysis were independently entered in an Excel spreadsheet and only shared with the results collation officer after finalizing.

### Power calculations and statistical analyses.

For the cross-sectional evaluation of the Schistoscope, the number of positive cases needed to achieve an assumed sensitivity and specificity of 80% and 90% using conventional microscopy as the reference was calculated to be 107.^23^ The power of this calculation was set to 80%, and a 5% degree of error was considered to be able to detect a difference of at most 10% from the assumed sensitivity and specificity. With a schistosomiasis prevalence of 25% in the FCT region,^19^ a total of 450 samples was needed to meet our target case number. Microscopy and Schistoscope data were merged and double-checked by the collation officer. Descriptive statistics for the data were obtained using IBM Statistical Package for Social Sciences version 25 (SPSS Inc., Chicago, IL). For the baseline sample subset, sensitivity, specificity, positive predictive value (PPV), and negative predictive value (NPV) of the semi- and fully automated digital microscope were calculated for *S. haematobium* detection using conventional light microscopy as the reference standard. Qualitative agreement between the Schistoscope and conventional microscopy was assessed using the adjusted Cohen’s kappa, considering true positives and true negatives, as well as false positives and false negatives.^24^ Egg counts were categorized as low-intensity infection (≤ 50 eggs/10 mL urine) or high-intensity infection (> 50 eggs/10 mL urine). Because of the non-Gaussian nature and wide range of the egg count estimates for all three methods, the data set was log transformed before analysis was performed. The linear association in terms of egg counts (eggs/10 mL) between the different optical procedures was estimated using the Pearson’s correlation coefficient (*r*), excluding the negative data points. Bland–Altman analysis was performed for quantitative assessment of the agreement between semi- and fully automated digital microscopy and conventional microscopy using GraphPad Prism version 9.0.1 for windows (GraphPad Software, San Diego, CA; http://www.graphpad.com). Cure rate (CR), defined as the percentage of follow-up samples with no detectable eggs, and egg reduction rate (ERR), defined as the percentage reduction in the geometric mean (GM; formula: GM (egg count +1) – 1) egg counts pre- and post-treatment, were estimated for each of the microscopy procedures.

## RESULTS

### Performance evaluation of the Schistoscope and estimation of egg counts.

To evaluate the capacity of the Schistoscope to detect and count *S. haematobium* eggs, each of the 487 prepared slides was examined by conventional microscopy and by both semi- and fully automated digital microscopy. No differences resulting from the order in which the filters were examined were noted (e.g., first by conventional microscopy, followed by image capturing by the Schistoscope or vice versa). The three detection methods (i.e., conventional and semiautomated and fully automated digital microscopy) independently identified 166 (34.1%), 148 (30.4%), and 309 (63.4%) of the slides as positive for *S. haematobium,* respectively (Table 1). Egg count estimates per 10 mL of urine ranged from 1 to 4,386 eggs/10 mL for conventional microscopy, 1 to 2,059 eggs/10 mL for semiautomated digital microscopy, and 1 to 573 eggs/10 mL for fully automated digital microscopy, with a median of 12, 12, and 2 eggs/10 mL, respectively. Compared with conventional microscopy, semi- and fully automated digital microscopy showed an overall accuracy of 90.1% and 62.0%, respectively (Table 1).

**Table 1:** Cross tabulation of the detection of *Schistosoma haematobium* eggs by the Schistoscope 5.0 and conventional microscopy performed on 487 urines collected at baseline screening

		Conventional microscopy		
Schistoscope 5.0		Positive (*n* = 166)	Negative (*n* = 321)	Total (*N* = 487)
Semi-automated digital microscope	Positive	133	15	148
	Negative	33	306	339
Fully automated digital microscope	Positive	145	164	309
	Negative	21	157	178

Table 2 summarizes the sensitivity, specificity, PPV, and NPV for the semi- and fully automated digital microscopy. The sensitivities of the semi- and fully automated digital microscope for the detection of *S. haematobium* eggs were comparable; 80.1% (95% confidence interval [CI]: 73.2–86.0%) and 87.3% (95% CI: 81.3–92.0%), respectively, but the fully automated procedure showed a much lower specificity (48.9%; 95% CI: 43.3–55.0%) than the semiautomated procedure (95.3%; 95% CI: 92.4–97.4%). This resulted in a low PPV (46.9%) for the fully automated digital microscope (Table 2).

**Table 2:** Diagnostic performance of the Schistoscope 5.0 for the detection of *Schistosoma* eggs performed on 487 urines collected at baseline screening

Conventional microscopy	Schistoscope 5.0							
	Semiautomated digital microscopy				Automated digital microscopy			
	Sen (95% CI)	Spec (95% CI)	PPV (95% CI)	NPP (95% CI)	Sen (95% CI)	Spec (95% CI)	PPV (95% CI)	NPV (95% CI)
All samples with *S. haematobium* infection (*N* = 166)	80.1 (73.2–86.0)	95.3 (92.4–97.4)	89.8 (84.0–94.2)	90.3 (87.0–93.2)	87.3 (81.3–92.0)	48.9 (43.3–55.0)	46.9 (41.2–53.0)	88.2 (83.0–93.0)
Low-intensity infection* (*n* = 129)	75.2 (67.0–82.3)	–	–	–	83.7 (76.1-90.0)	–	–	–
High-intensity infection† (*n* = 37)	97.3 (86.0–100.0)	–	–	–	100	–	–	–

CI = confidence interval; NPV = negative predictive value; PPV = positive predictive value; Sen = sensitivity; Spec = specificity.

* ≤ 50 eggs/10 mL urine.

† > 50 eggs/10 mL urine.

Conventional microscopy classified 129 (78%) as low-intensity infection and 37 (22%) as high-intensity infection, whereas semi- and fully automated microscopy classified 111 (75%) and 294 (95%) as low-intensity infection and 37 (25%) and 15 (5%) as high-intensity infection, respectively. The sensitivities of semi- and fully automated digital microscopy for low-intensity infections were 75.2% (95% CI: 67.0–82.3%) and 83.7% (95% CI: 76.1–90.0%), which increased for high-intensity infections (Table 2). The adjusted Cohen’s kappa demonstrated a fair (0.34) and a slight (0.2) qualitative agreement between conventional microscopy and semi- and fully automated digital microscopy, respectively.

**Figure 3.**
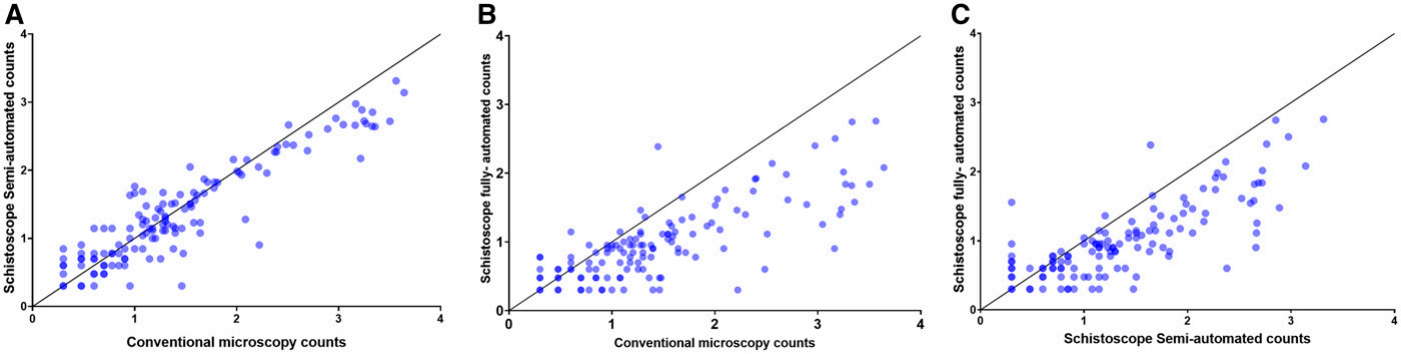
Correlation in *Schistosoma haematobium* egg counts per 10 mL of urine on a Log10 scale on samples collected at baseline screening. Negative data points are excluded. (**A**) Semiautomated digital microscopy versus conventional microscopy (*n *= 133, *r* = 0.90, *P* < 0.001). (**B**) Fully automated digital microscopy versus conventional microscopy (*n* = 145, *r* = 0.80, *P* < 0.001). (**C**) Semiautomated versus fully automated digital microscopy (*n* = 137, *r* = 0.80, *P* < 0.001). The depicted solid line indicates y = x. This figure appears in color at www.ajtmh.org.

**Figure 4.**
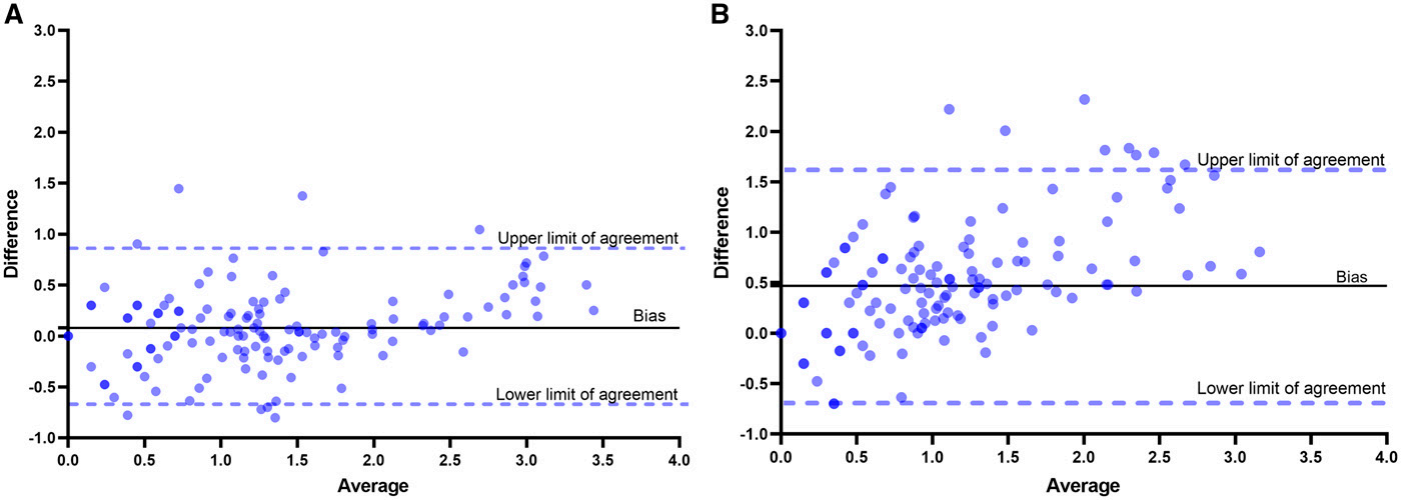
Bland–Altman plots showing the level of agreement between (**A**) conventional microscopy and semiautomated digital microscopy counts and (**B**) conventional microscopy and fully automated digital microscopy counts. This figure appears in color at www.ajtmh.org.

In terms of *S. haematobium* egg count estimates, conventional microscopy correlated strongly to semiautomated digital microscopy (*N* = 133, *r* = 0.90, *P* < 0.001) and fully automated digital microscopy (*N* = 145, *r* = 0.80, *P* < 0.001) (Figure 3). To demonstrate reliability of conventional microscopy, Bland–Altman analysis showed a strong agreement between the first and second microscopy readings across the range of mean egg counts for both readings (bias = 0.13, 95% limits of agreement from –0.66 to 0.94). Further Bland–Altman analysis demonstrated a strong agreement between conventional microscopy and semiautomated digital microscopy across the range of mean egg counts for both methods (bias = 0.08, 95% limits of agreement from –0.69 to 0.85) (Figure 4). Conventional microscopy and fully automated digital microscopy revealed a strong agreement at low mean egg counts of both methods. However, an underestimation of egg counts by fully automated digital microscopy was observed at egg counts greater than 100 eggs/10 mL (bias = 0.47, 95% limits of agreement from –0.69 to 1.63).

### Follow-up after praziquantel treatment.

Conventional microscopy and the semiautomated Schistoscope procedure were also compared on 38 urine samples collected 10 days post-praziquantel treatment from participants with a confirmed infection at baseline. Thirty (79%) and 27 (71%) samples still had detectable *S. haematobium* eggs by conventional microscopy and semiautomated digital microscopy, resulting in a CR of 21% (95% CI: 10–37) and 29% (95% CI: 15–46), respectively. In four follow-up samples, eggs were only seen by conventional microscopy, and only one sample was positive by semiautomated digital microscopy. The ERR of conventional microscopy (80%; 95% CI: 64–90) and semiautomated digital microscopy (77%; 95% CI: 60–91) were similar.

## DISCUSSION

In this study, the performance of the Schistoscope 5.0 was evaluated as a semiautomated digital microscope and as an AI-based fully automated digital microscope for the detection and quantification of *S. haematobium* eggs in a field setting. The diagnostic parameters that were assessed include sensitivity, specificity, PPV, NPV, and infection intensity. At baseline screening, the sensitivity of the semiautomated digital microscope (80.1%) was lower than that of the fully automated digital microscope (87.3%); however, this difference was not statistically significant. As expected, the sensitivity of the Schistoscope increased with increasing egg excretion. On the other hand, the Schistoscope detected additional cases as positive, which might have been true cases missed by conventional microscopy. Conventional microscopy was used as the standard reference, and this resulted in a reduced specificity of the Schistoscope. The specificity was significantly lower for the fully automated digital microscope (48.9%) than for the semiautomated digital microscope (95.3%).

A probable reason for the low specificity recorded by the fully automated digital microscope is the limited datasets used to train the AI algorithm to detect *S. haematobium* eggs. The AI algorithm was developed using two training datasets consisting of images obtained from egg-spiking experiments resulting in relatively clean samples and a limited number of field samples that did not contain many egg-like artifacts (e.g., uric crystals). Therefore, the AI algorithm seemed insufficiently trained to separate egg-like artifacts from *S. haematobium* eggs. Another reason could be limitations in the deep learning model used by the AI algorithm that was optimized for enhanced sensitivity at a trade-off of specificity. Additional iterations to enhance specificity are therefore needed and are currently in progress.

Several other studies have also field evaluated digital optical devices, with or without AI, for the detection and/or quantification of *S. haematobium* eggs.^9^^,^^11^^,^^12^ The sensitivities and specificities obtained for the various devices in these studies, with conventional microscopy as a reference, range from 35.6% to 81.1% and 91.0% to 100%, respectively. The sensitivities of the semi- and fully automated digital microscope reported in the current study were generally higher compared with previous reports, except for results reported by Coulibaly et al., for the Newton Nm1 microscope, which is considered comparable in sensitivity. However, the study by Coulibaly et al. had a slightly lower power than our study, with 266 samples examined, of which 90 were egg positive.

For egg count estimates, a strong correlation was observed between semiautomated digital microscopy and conventional microscopy, whereas for fully automated digital microscopy, a clear underestimation of the intensity of infection was observed for samples with more than 100 eggs/10 mL urine. A possible explanation is that overlapping eggs were recognized as a single egg by the deep learning model, leading to an underestimation of egg counts. In addition, hematuria might have also caused interference. Although not systematically recorded, our impression was that samples with more than 100 eggs/10 mL of urine were often strongly positive for hematuria, with an abundance of blood cells compared with samples with lower egg counts. This could have resulted in shading the eggs on the filter membrane and subsequently limiting the detection by the AI algorithm.

Although only performed in a small subset of cases and at one time point, no substantial differences were noticed before and after treatment when comparing the semiautomated digital microscope with the conventional microscopy, suggesting that the Schistoscope could also be used for monitoring treatment. More extensive posttreatment follow-up studies are needed to demonstrate how well the Schistoscope can differentiate viable *S. haematobium* eggs from dead eggs, which can be excreted up to many weeks after receiving praziquantel (personal observation).

The Schistoscope 5.0 captured high-resolution images that clearly show the specific features of *S. haematobium* and *S. mansoni* eggs (i.e., the terminal and lateral spines; Figure 1C). In terms of potential use-cases, this supports the application of the semiautomated microscope as a diagnostic tool to assist microscopists in field laboratory settings. The use of (semi-)automated digital microscopy could reduce visual health complications caused by high eye exposure to a conventional microscope light source. Upon further development to improve the AI, the fully automated microscope would be useful for nonexpert microscopists as well (e.g., community health workers and laboratory technicians). In both cases, task shifting could be gained because personnel could focus on other activities while the device analyzes samples. The added value of task shifting could compensate for the current time difference between conventional microscopy that requires less than 10 minutes to scan a urine filter and the Schistoscope 5.0, which can take on average 17 minutes to complete scanning and analysis.

Limitations of this study include the choice of conventional light microscopy on a single 10 mL urine sample as the reference test, which is known for its limited sensitivity, especially in cases with low infection intensity. Further evaluation studies should be conducted to field validate the Schistoscope 5.0 for the detection of *S. haematobium* eggs compared with more sensitive reference tests such as the detection of adult worm-associated circulating anodic antigens or the detection of parasite specific DNA.^25^ The Schistoscope 5.0 currently does not meet the target product profile set by the WHO for new diagnostics needed for monitoring and evaluating schistosomiasis control programs.^26^ For example, it does not have an onboard display and is connected to a computer monitor for visual control of the device, thus making transportation impractical. Furthermore, the device lacks a backup power supply. Additional functionalities such as an onboard computer with a graphical processing unit for higher image processing capabilities and internet access would also be beneficial. These functionalities would create the capacity to generate results in real time for patient management, store and share digital images with other experts, and facilitate mapping of schistosomiasis,^27^ thereby making (semi-)automated digital devices an attractive tool for future use in epidemiology and public health settings. Here we evaluated the Schistoscope 5.0 for the first time in a rural field setting, demonstrating its potential as a digital diagnostic tool for the detection and quantification of *S. haematobium* eggs, as well as for monitoring the effect of schistosomiasis treatment in settings with limited resources.
